# Assessment of interventricular systolic relationship and infarct location in acute myocardial infarction

**DOI:** 10.1186/1532-429X-17-S1-P21

**Published:** 2015-02-03

**Authors:** Pankaj Garg, Ananth Kidambi, Laura E Dobson, Tarique A Musa, David P Ripley, Adam K McDiarmid, Bara Erhayiem, Peter P Swoboda, John P Greenwood, Sven Plein

**Affiliations:** 1 Multidisciplinary Cardiovascular Research Centre & Leeds Institute for Cardiovascular and Metabolic Medicine, University of Leeds, Leeds, UK

## Background

Longitudinal motion of the mitral valve, known as the mitral annular plane of systolic excursion (MAPSE), correlates well with LV systolic function. Similarly, tricuspid annular plane systolic excursion (TAPSE) correlates well with RV systolic function. Echocardiographically, the absolute value of TAPSE was greater than MAPSE, by 54.5% in a recent study of normal subjects and the MAPSE/TAPSE ratio is 0.66±0.14 (1). However, the interventricular systolic relationship has not been accurately defined in the presence of regional wall motion abnormalities. We hypothesised that the interventricular systolic relationship, measured by the ratio of averaged-MAPSE and TAPSE, changes in the presence of acute myocardial infarction with regional wall motion abnormality.

## Methods

**Figure 1:**
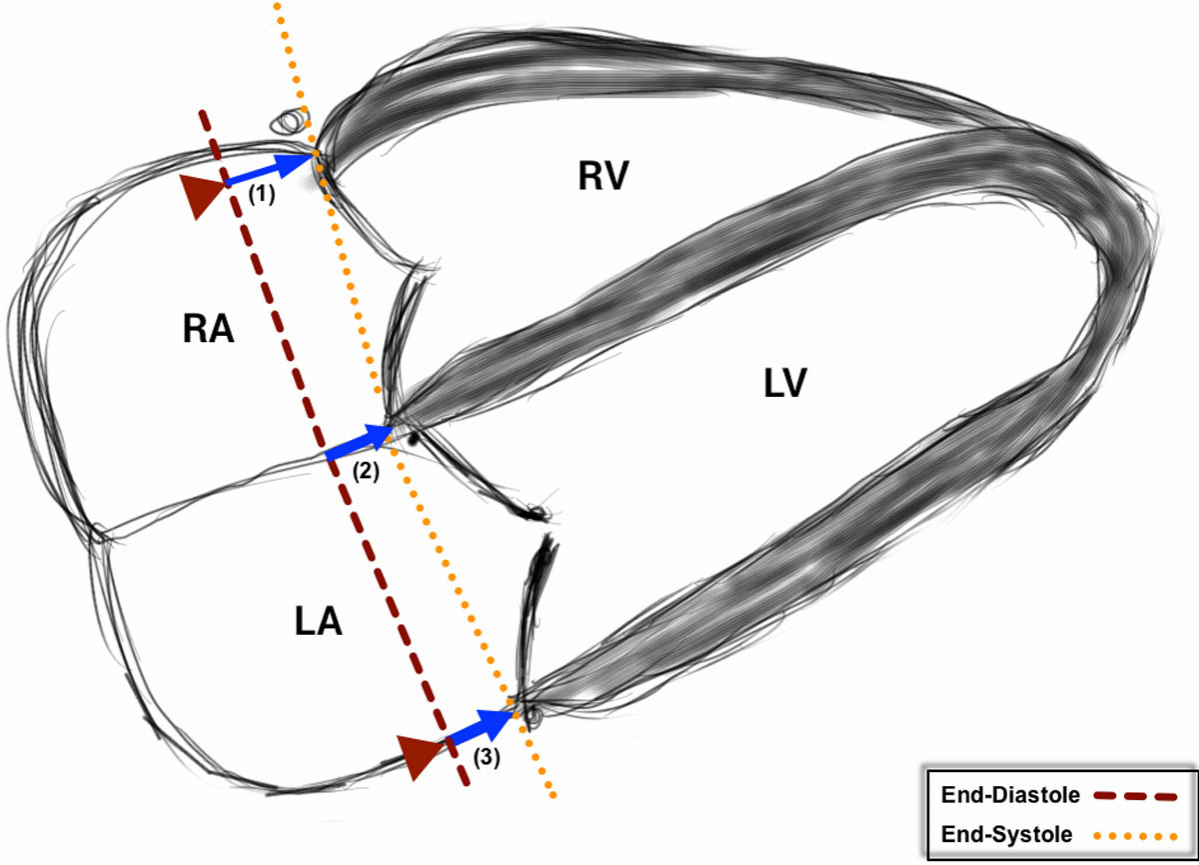
Diagram showing how the three longitudinal parameters were acquired. (1- TAPSE; 2- Medial MAPSE; 3- Lateral MAPSE)

Thirty-eight patients underwent CMR at 3T (Intera CV, Philips Healthcare, Best, The Netherlands) within 3 days following reperfused ST-elevation MI. Black blood, cine and LGE imaging (16-20 minutes following 0.1 mmol/kg gadolinium DTPA) were performed. Infarct location (anterior; lateral; inferior) was determined by location of LGE in the infarcted area. MAPSE (medial, lateral and average) was measured from the 4-chamber cine. A line was drawn across both the medial and lateral mitral annulus as a reference point in end-diastole (just after closure of the mitral valve). A second reference line was drawn across the same plane on an image taken just after closure of the aortic valve. The longitudinal distance parallel to the left ventricular wall was measured for both medial and lateral walls. Using a similar technique, TAPSE was measured (Figure 1). The infarct volume was measured from LGE images by semi-automated thresholding (Otsu method).

## Results

Patient characteristics and basic CMR results are listed in Table 1. The absolute value of TAPSE was 54.5% greater than the MAPSE value; comparable to values reported previously in normal populations. The MAPSE/TAPSE ratio in acute-MI patients was significantly reduced compared to reported normal values (0.56±0.14 vs 0.66±0.14; p=0.003). LV ejection fraction (EF) correlated most closely with averaged MAPSE (r=0.45; p=0.004). Infarct location had no significant influence on the LVEF (p=0.26) and on the interventricular systolic relationship measured by the ratio of averaged-MAPSE/TAPSE (r=-0.14; p>0.05). The interventricular systolic relationship significantly correlated with left ventricular end-diastolic indexed volume (LVEDVi)(r=-0.46; p=0.009) and infarct volume (r=-0.36; p=0.04).

**Table 1:** Basic CMR values (mean and standard deviation (SD)) for the four-infarct zones.

	Anterior MI (n=20)	Lateral MI (n=6)	Inferior MI (n=12)	p-value
EF (%)	44.6±10.4	51.1±10.2	48.9±8.3	0.26

Lateral MAPSE (mm)	10.92±2.2	11.7±3.0	10.36±2.2	0.53

Medial MAPSE (mm)	9.11±3.0	10.3±2.7	10.14±3.0	0.55

TAPSE (mm)	19.30±5.2	19.44±3.5	21.17±5.9	0.60

Averaged MAPSE (mm)	10.02±2.0	10.97±1.6	10.25±2.3	0.65

MAPSE/TAPSE	0.53±0.1	0.58±0.1	0.50±0.1	0.29

Infarct Volume (mls)	14.4±12.0	19.31±12.3	14.3±14.8	0.69

LVEDVi (ml/m2)	83.9±14.0	73.6±16.1	84.4±18.4	0.34

Values expressed in Mean ± Standard Deviation(SD).

## Conclusions

The ratio of averaged-MAPSE/TAPSE is significantly reduced in patients with acute-MI and correlates with infarct size. In cases where acute-MI is suspected, this parameter of interventricular systolic relationship may provide a simple additional diagnostic tool for both echocardiography and CMR assessment.

## Funding

JPG and SP receive a research grant from Philips Healthcare. SP is funded by British Heart Foundation fellowship (FS/10/62/28409).
